# Regularized adversarial learning for normalization of multi-batch untargeted metabolomics data

**DOI:** 10.1093/bioinformatics/btad096

**Published:** 2023-02-24

**Authors:** Andrei Dmitrenko, Michelle Reid, Nicola Zamboni

**Affiliations:** ETH Zürich, Institute of Molecular Systems Biology, Zürich 8093, Switzerland; Life Science Zurich PhD Program on Systems Biology, Zurich, Switzerland; ETH Zürich, Institute of Molecular Systems Biology, Zürich 8093, Switzerland; ETH Zürich, Institute of Molecular Systems Biology, Zürich 8093, Switzerland; PHRT Swiss Multi-OMICS Center, Switzerland

## Abstract

**Motivation:**

Untargeted metabolomics by mass spectrometry is the method of choice for unbiased analysis of molecules in complex samples of biological, clinical or environmental relevance. The exceptional versatility and sensitivity of modern high-resolution instruments allows profiling of thousands of known and unknown molecules in parallel. Inter-batch differences constitute a common and unresolved problem in untargeted metabolomics, and hinder the analysis of multi-batch studies or the intercomparison of experiments.

**Results:**

We present a new method, Regularized Adversarial Learning Preserving Similarity (RALPS), for the normalization of multi-batch untargeted metabolomics data. RALPS builds on deep adversarial learning with a three-term loss function that mitigates batch effects while preserving biological identity, spectral properties and coefficients of variation. Using two large metabolomics datasets, we showcase the superior performance of RALPS as compared with six state-of-the-art methods for batch correction. Further, we demonstrate that RALPS scales well, is robust, deals with missing values and can handle different experimental designs.

**Availability and implementation:**

https://github.com/zamboni-lab/RALPS.

**Supplementary information:**

Supplementary data are available at *Bioinformatics* online.

## 1 Introduction

Metabolomics is the method of choice for chemical characterization of biological, clinical and environmental samples (Alseekh *et al.*, 2021; Johnson *et al.*, 2016; Patti *et al.*, 2012). When the aim of the analysis is to monitor many and potentially unexpected analytes, the traditional untargeted approach is to scan the full mass and dynamic range with high-resolving instruments. This strategy allows monitoring of virtually all compounds that can be ionized and are sufficiently abundant. Eventually, untargeted metabolomics experiments result in semi-quantitative data for thousands of detectable features and many more unknowns. A largely unsolved problem of such large metabolomics experiments, however, is data normalization. The sheer number of features and the extreme sensitivity of liquid chromatography–mass spectrometry (MS) instruments to multiple factors mean that ion-specific temporal drifts, day-to-day variability or inter-batch effects are common in metabolomics analyses (Alseekh *et al.*, 2021; Dunn *et al.*, 2011). These problems increase with number of samples.

The most common way to account for and correct these issues is to employ isotopically labeled internal standards, which are added at a fixed amount to all samples and calibration standards (Patti *et al.*, 2012; Schatschneider *et al.*, 2018; Sysi-Aho *et al.*, 2007; Wu *et al.*, 2005). If the standard’s elution times and ionization behavior are identical to those of the compound of interest, the standard can be used to correct for linear matrix effects. This approach is quite effective with a limited number of metabolites, as in targeted metabolomics analyses, but does not scale to untargeted metabolomics. The first problem is the limited availability of heavy standards, and one workaround is to use a few representatives for each class and extrapolate over structurally similar compounds. The second problem is the limit on the number of standards that can be spiked without introducing novel matrix effects, i.e. when using standards available in salt form.

In the absence of internal standards to assess and correct for experimental variations, drifts, batch effects and so on, normalization needs to operate on the resulting data. Intra-batch ionization drifts are effectively corrected by detrending on the basis of quality control samples (Broadhurst *et al.*, 2018; Dunn *et al.*, 2011; Kuligowski *et al.*, 2015; Rusilowicz *et al.*, 2016). Inter-batch biases, however, are more challenging, and many different approaches to reduce them have been presented in recent years. Notable examples of inter-batch normalization methods are ComBat (Johnson *et al.*, 2007), based on empirical Bayes frameworks and EigenMS (Karpievitch *et al.*, 2014), using matrix factorization by singular value decomposition. Together with probabilistic quotient normalization (PQN) (Dieterle *et al.*, 2006), these approaches have been reported as some of the best options for untargeted metabolomics studies (Yang *et al.*, 2020) when applied in combination with metabolite-based scaling (e.g. LEV+EIG for level scaling followed by EigenMS, or PQN+POW for PQN followed by power scaling). In 2019, an algorithm based on the wavelet transform with independent component analysis, WaveICA, was introduced and showed superior performance in large-scale untargeted metabolomics studies (Deng *et al.*, 2019).

A novel avenue for batch correction in metabolomics was opened by NormAE (Rong *et al.*, 2020). It employs deep learning and was inspired by the very successful application in single-cell RNA sequencing (Lakkis *et al.*, 2021; Li *et al.*, 2020; Wang *et al.*, 2019). NormAE relies on adversarial learning in which two neural networks, an autoencoder and a classifier, are trained simultaneously to reconstruct the data and classify batches based on the latent space of the autoencoder, respectively. The ultimate goal is to reproduce the data but remove differences between user-defined batches. To achieve this, the autoencoder is trained with a loss function that includes two terms. The first term awards correct reconstruction, and the second term applies a penalty if samples from different batches are separated correctly. The latter term pushes the autoencoder to learn the data representations that render any user-defined batches indistinguishable from each other. NormAE set new performance standards in removing batch effects (Rong *et al.*, 2020) but still suffers from issues common to all practical applications of deep learning, such as the non-trivial parameter optimization, computational complexity and lack of reproducibility (Bhojanapalli *et al.*, 2021). Further, it requires identical reference samples across all batches, which might become a problem in long-term studies. Finally, NormAE and some other aforementioned approaches produce heavily rescaled normalized outputs in arbitrary units, undermining interpretability.

Here, we present a new normalization method, Regularized Adversarial Learning Preserving Similarity (RALPS), for untargeted metabolomics that efficiently addresses all the aforementioned problems. RALPS builds on adversarial learning but implements a novel three-term loss function that suppresses batch effects while preserving biological information. Using several test sets, we show that RALPS outperforms state-of-the-art methods in terms of performance, scalability, usability and robustness.

## 2 Results

### 2.1 Method overview

**Fig. 1.**
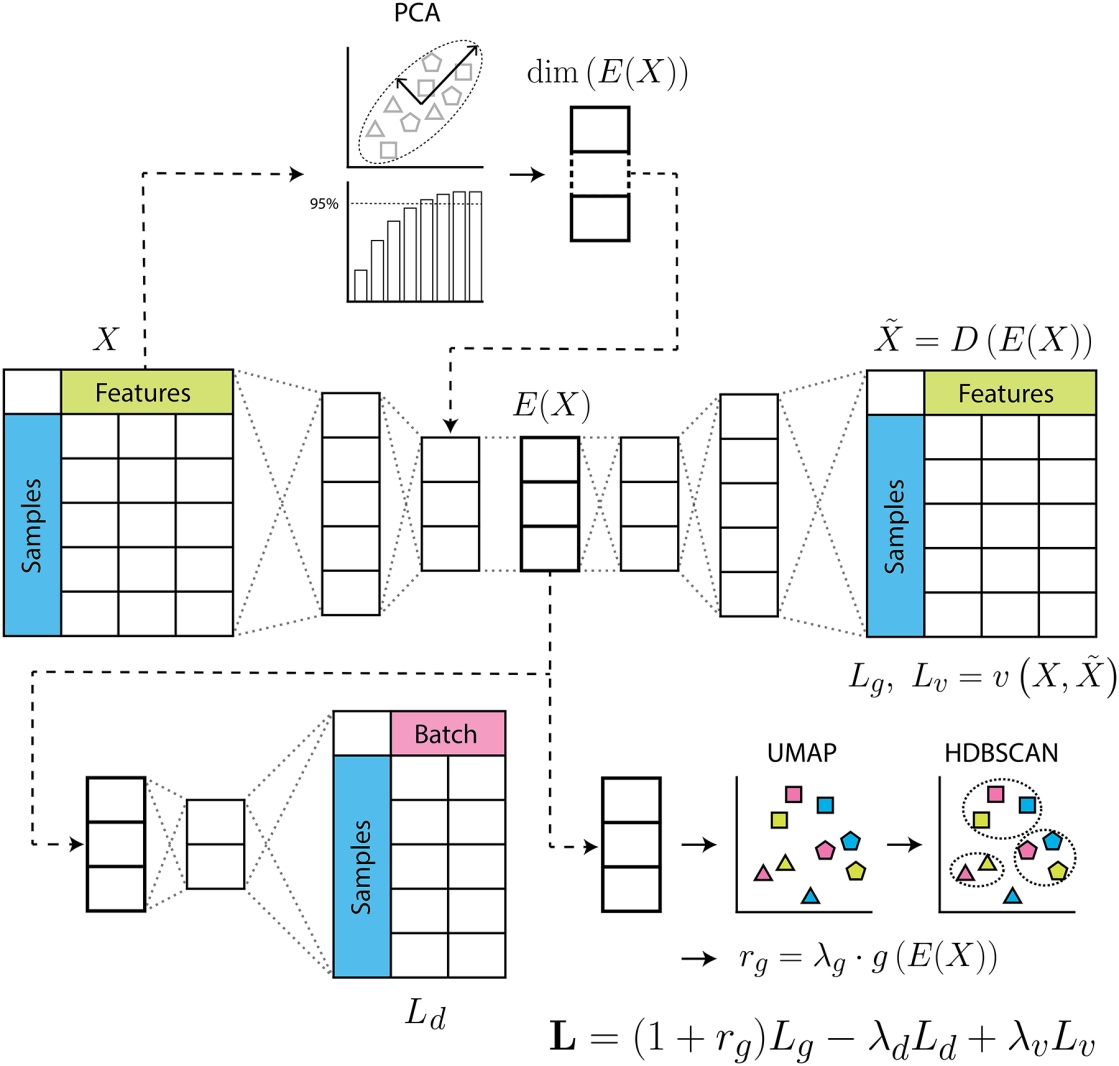
Graphical overview of RALPS. The autoencoder takes measured data, *X*, as input and produces its reconstruction, *D(E(X))*, as output. The number of neurons in the bottleneck layer is determined by principal component analysis. The aggregated autoencoder loss **L** consists of three terms: the regularized autoencoder loss *L_g_*, the classifier loss *L_d_* and the variation loss *L_v_* with real coefficients *λ_g_*, *λ_d_* and *λ_v_*, respectively

RALPS was inspired by NormAE: it uses an autoencoder and a classifier to mitigate batch effects, but the loss function was extended with two additional terms beyond the reconstruction loss (*L*_g_) and batch discrimination loss (*L*_d_) (Fig. 1). First, we wanted to introduce a mechanism to preserve characteristic differences of any set of supposedly similar samples across the whole sequence as a way to preserve biological information. For this purpose, we added a new regularization term (*r*_g_) to the autoencoder loss function to reward tight clustering of reference samples in the embedded space. To evaluate clustering, RALPS flattens the multidimensional space by uniform manifold approximation and projection (UMAP) (McInnes *et al.*, 2018) and performs unsupervised hierarchical density-based cluster analysis (HDBSCAN) (Malzer and Baum, 2020). Eventually, clustering is evaluated by counting how frequently reference samples of the same type occur in the same cluster. In the case of perfect clustering of biological replicates, the regularization term is zero. We also included a new variation loss term (*L*_v_) to encourage a decrease in batch variation coefficients (VCs). This addition was motivated by two observations. First, many normalization methods tend to inflate the VCs of replicate measurements. Second, deep-learning models with encoder–decoder architectures are especially prone to producing outliers.

Furthermore, we included several additional features to improve scalability, usability and robustness. First, RALPS adopts a flexible network architecture, in which the number of neurons in the model layers is automatically adjusted based on a principal component analysis. By default, RALPS sets the number of neurons equal to the number of principal components needed to describe at least 90% of the dataset variance. This modification eliminates many hyperparameters from the model and simplifies parameter optimization. Second, we introduced a randomized hyperparameter grid search and model selection logic, both of which enable finding multiple parameter sets that deliver top normalization results in an automated way. Third, we introduced input validation and a mechanism for early stopping to avoid collapsed normalization solutions that could arise because of inconsistent parametrization or increasing classifier loss. Details are provided in the Supplementary Material. RALPS is implemented in Python programming language with PyTorch deep-learning framework. It requires a single configuration file containing the data and the batch information file paths, as well as a few other parameters to run normalization.

Information on reference samples is provided by the user to indicate all sample groups that are supposed to be similar. These groups can include biological replicates, technical replicates, pooled study samples, spike-ins and dilution series. There is no formal limit on the number of reference groups, and although sample groups should span multiple batches, the same reference samples do not need to be present in all batches. This flexibility builds considerable freedom into the experimental design, as discussed below.

### 2.2 Generation of a multi-batch benchmarking dataset

We faced the problem of finding suitable multi-batch datasets for testing and comparing normalization methods. Because such datasets are rare and often associated with clinical studies that preclude full disclosure and publication (Rong *et al.*, 2020), we opted to generate a novel benchmarking dataset. We assembled a panel of 136 samples with a large variety in sample type, complexity and concentrations (Supplementary Methods and Supplementary Fig. S1). Roughly half of the samples were prepared in human serum extracts, the rest in water. We included spike-ins with selections of amino acids, fatty acids and nucleobases, and a fully ^13^C-labeled *Escherichia coli* extract at different dilutions. The samples were aliquoted, frozen and analyzed seven times over the span of about 2 months with time gaps of up to 3 weeks. Each of the seven batches was acquired and processed independently. To focus on the subset of m/z peaks that possibly relate to metabolites, we selected features with m/z values that were matched to deprotonated compounds listed by the Human Metabolome Database (ver. 4.0, tolerance 0.001 Da). Upon intersecting the putative peak lists obtained from each batch and filtering low-abundance features, we obtained a data table with intensities for 170 putative deprotonated metabolites and 2856 files, divided in seven batches.

To visualize the extent of batch effects, we plotted the UMAP projections for all samples (Supplementary Fig. S2a). We observed that samples grouped by batch, and almost no overlap between batches could be found. This result clearly indicates that batch effects dominate and confound chemically identical samples. Another way to quantify batch effects is to calculate the cross-correlation of identical samples within and between batches (Supplementary Fig. S2b). Although Pearson correlation coefficients were >0.9 within the same batch, the distribution of inter-batch correlations shows a heavy tail, reaching values as low as *r* = 0.4, pointing to strong biases. Below, we use this dataset to benchmark RALPS against state-of-the-art approaches.

### 2.3 Normalization of the multi-batch dataset

As a first demonstration, we evaluated RALPS on a multi-batch benchmarking dataset. Initially, all sample labels were included as reference groups to maximize information available for training. We ran RALPS with default parameters and a randomized grid search of size 50. The best normalization solution selected by the model selection logic was compared to the initial dataset. First, we assessed the normalization effect of our method by highlighting batch labels on the UMAP embeddings plot (Supplementary Fig. S2d). We observed that replicates from different batches were largely mixed for the normalized data compared with the initial data. The distribution of cross-correlations of all samples’ replicates within and between batches shifted close to the ideal value of one (Supplementary Fig. S2e). Moreover, VCs calculated for all intensities in every batch were consistently reduced in the normalized data (Supplementary Fig. S2f) compared with the initial data (Supplementary Fig. S2c). The total runtime for the 50 independent runs was 291 min, i.e. about 6 min per run for a dataset with almost 3000 samples. Note that only a single CPU core was used for all computations. With an increasing number of CPU cores, it would be possible to evaluate much larger hyperparameter sets overnight, increasing the probability of finding an optimal normalization solution.

### 2.4 Normalization of the multi-batch dataset with limited reference groups

The test described above builds on a rather artificial scenario in which all samples are present in every batch. In practice, however, only a small subset of samples in each batch are repetitions of samples of different batches and can be used to correct for inter-batch effects. The most common scenario is inclusion of one or two reference samples in all batches. If a single sample is available, a frequent option is to spike in standards at a relevant concentration to ensure correct recovery of significant differences, including MS measurement and data processing. To mimic this realistic scenario, we attempted to normalize the benchmarking dataset with RALPS, using only a few reference sample groups each time. Different sets of groups were tested. For instance, Supplementary Figure S3 shows the normalization achieved by RALPS using exclusively an undiluted NIST1950 serum extract (P2_S_0001) and the same sample spiked with purines and pyrimidines (P2_S_PP_0001). The labels and relation of all remaining samples were neglected during training. For the normalized data, batches no longer appeared as isolated clouds of points, and instead, samples from different batches were mostly well mixed. Qualitatively, the result was similar to the initial test in which all reference groups were provided during the training phase.

To demonstrate the flexibility of RALPS, we tested several combinations of up to four reference sample groups. For each combination, we applied RALPS with a randomized grid search of size 100. We used default parameters and set *λ*_v_ = 0 to loosen the constraints on the joint loss function optimized during training (Fig. 1).

We found several combinations of reference samples that produced good normalization effects (Supplementary Table S1). Comparing some of the evaluation metrics, using only undiluted NIST1950 serum extract (P2_S_0001) or using combinations of two or three samples yielded results similar to those from the examples described above (row #1 in Supplementary Table S1). As a negative control, we provide results obtained by training with a single reference group consisting of highly diluted fatty acids in water (row #10 in Supplementary Table S1). Because of matrix differences and the common presence of fatty acids in the background, these samples are unlikely to be sufficient to correct for complex batch effects, e.g. in serum samples.

All other tested configurations were comparably superior on all evaluation metrics, but not all were reproducible, as assessed by several repetitions of the training with identical reference groups but different random starts. Only half of the cases frequently reproduced comparably good results. For the other half that was not reproducible, including the case with undiluted serum as a unique reference group, multiple independent attempts would be necessary. Nevertheless, all the reference group configurations performed well in mixing batches on the UMAP embedding plot (Supplementary Fig. S4). These results prove that RALPS is flexible in the choice of reference samples and, in principle, a few reference sample groups in triplicate can suffice.

**Fig. 2.**
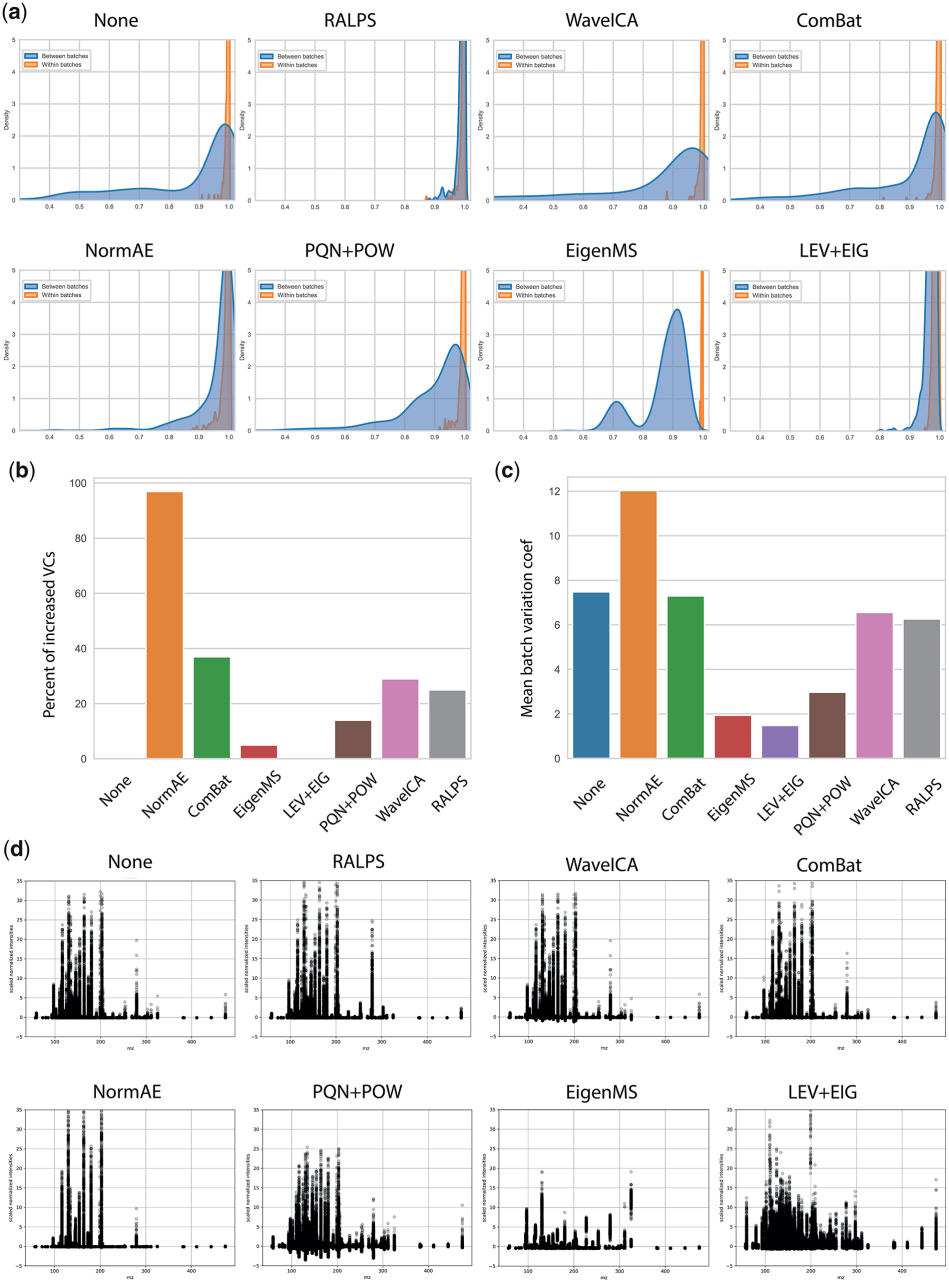
Comparison of methods for the benchmarking dataset. (**a**) Distributions of cross-correlation of reference samples within batches and between batches. The correlation was calculated for all samples of the same type. (**b**) Percent of samples with increased VCs upon normalization. (**c**) Mean batch VCs. (**d**) Centroided spectra. For comparison, initial and normalized peak intensities (*y*-axis) were scaled by *z*-score

Next, we compared RALPS to the state-of-the-art normalization approaches mentioned in the introduction. Of the several training scenarios listed above, RALPS was trained using two reference sample groups with undiluted serum and the derivative with spiked purines and pyrimidines (Supplementary Fig. S3). The performance was evaluated using one qualitative and three quantitative criteria. The quantitative criteria included cross-correlation of all samples’ replicates, batch VCs and percent of features for which the VC across replicate samples increased upon normalization. The qualitative criterion was the spectrum of the normalized data compared with that of the initial data (Fig. 2).

We found that three of seven methods improved the cross-correlation of replicates among batches: RALPS, NormAE and LEV+EIG (Fig. 2a). In Supplementary Figure S5, the effect of normalization on cross-correlation is shown in detail for one of the samples that featured prominent bias effects, i.e. P1_FA_0008. This sample was an 8-fold diluted fatty acid mix that was strongly affected by the common background signal of fatty acids. For this example, RALPS and LEV+EIG achieved the best results, with correlation coefficients >0.9. In the case of RALPS, a small bias between the first batch and all others remained. A different picture was obtained in terms of improving mean batch VCs. All methods except NormAE resulted in reduced mean batch variance (Fig. 2b). However, LEV+EIG, EigenMS and PQN+POW each resulted in a drastic drop that suggests a degenerated solution. In practice, in the attempt to reduce batch effects, these methods also may attenuate biological differences and obscure the information of interest.

The third quantitative evaluation criterion highlights a frequently overlooked caveat of normalization procedures: the fraction of sample whose VC increases by 5% or more upon normalization. The expectation is that normalization removes inter-batch biases without compromising intra-batch reproducibility. However, an apparent improvement in inter-batch reproducibility can also be attained by drastically worsening intra-batch precision, generally confounding all data and biological information. The criterion cutoff is arbitrary but provides a simple assessment for the frequency of variance inflation across features. We again found the best results with EigenMS, LEV+EIG and PQN+POW (Fig. 2c). NormAE, meanwhile, inflated the VC in >95% of the cases. RALPS fell in the middle average of the methods and outperformed ComBat and WaveICA. The striking difference between NormAE and RALPS highlights the relevance of the additional term *L*_v_ that was introduced in the loss function to control for an increase in variance.

Lastly, we examined the MS spectra obtained by normalization. From an analytical standpoint, the expectation is that normalization will have a minor effect on the overall spectrum. In reality, this is the case only if the ranking of metabolites based on average intensities is roughly maintained. In contrast, a normalization procedure that has a large effect on the value ranges of features will result in a qualitatively different spectrum. Spectra preservation is particularly important if calibrants are included to estimate concentrations. By visual inspection of the resulting spectra (Fig. 2d), we indeed observed that using methods with the lowest batch VCs (LEV+EIG and EigenMS) completely altered the data. NormAE preserved high-intensity peaks but suppressed low-intensity features, which is in agreement with the previously reported issue of sensitivity to outliers (Rong *et al.*, 2020). PQN+POW yielded the opposite pattern and amplified low-abundance ions. RALPS, WaveICA and ComBat had the most neutral impact on the spectra, preserving both high- and low-intensity features. Of note, WaveICA and ComBat, but not RALPS, can output negative ion intensity values, which can result in complications in downstream data analysis and interpretation. In summary, RALPS was the only method among the seven tested approaches to excel in the suppression of batch biases while controlling for mean batch variance, replicate VCs and drastic spectral transformation.

### 2.5 RALPS corrects biases on multi-batch cancer cell metabolomics data

We further tested the performance of RALPS with data published by Cherkaoui *et al.* (2021). This dataset includes >1400 untargeted metabolomics measurements for a panel of ca. 180 cancer cell lines, resulting in a matrix with relative abundances for 1817 putative metabolite ions. This dataset is interesting for three reasons. First, it represents real-life, mid-sized untargeted metabolomics studies. Second, its batch effects are associated with sample preparation and not with sample acquisition, as in the benchmarking dataset. This association is present because the limiting step in the study was sample generation and not MS analysis. Because of the tedious procedures necessary to cultivate numerous cell lines in parallel, the entire study was divided into seven batches of samples generated over the span of about a year. Upon preparation, cell pellets were stored at −80°C, and when the seven sampling batches were complete, all samples were prepared and subjected to sequential MS analysis. The expected batch effects thus are dominated by shifts in cultivation conditions (e.g. media, incubation conditions and handling). The third aspect of interest is that the study did not use a set of reference samples that were included in all batches. Only two cell lines (MDAMB231 and MCF7) were present in multiple batches (five and four, respectively) and were used as reference samples in the training phase of RALPS.

As NormAE failed to complete normalization with the full dataset, we focused the comparison on the subset of ca. 170 features that were putatively annotated as deprotonated metabolites. All methods seemingly improved cross-correlation of the MDAMB231 sample, except for PQN+POW (Supplementary Fig. S6a). Additional odd results included a mean batch variance that dropped to almost zero for LEV+EIG and almost doubled for NormAE (Supplementary Fig. S6d). In line with the previous results, spectra were altered by LEV+EIG, EigenMS, PQN+POW and NormAE. Overall, RALPS and WaveICA were the best methods for normalizing the data from Cherkaoui *et al.* ComBat was also a good alternative, but the final cross-correlations of MDAMB231 across samples were worse, and more control samples would be needed for more precise conclusions. Among the three best methods, RALPS also appeared to better preserve low-intensity data. ComBat and WaveICA produced negative values that affected ca. 1% of the samples (Supplementary Table S2). The normalized negative values exceeded 106 counts, resulting in obvious complications for further analysis and interpretation. In contrast, RALPS produced strictly positive values.

**Fig. 3.**
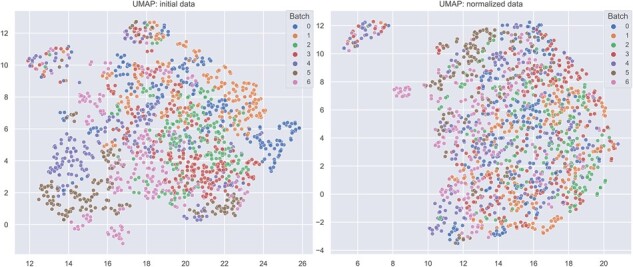
UMAP embeddings for the cancer cell lines dataset by Cherkaoui *et al.* Initial (left) versus normalized with RALPS (right) data is presented

Finally, we compared the evaluation times of all approaches (Supplementary Table S3). A single run of RALPS took about 3 min. A randomized grid search with 50 samples required about 2–3 h, which was similar to the time required by the slowest algorithms, such as NormAE (even using a GPU), EigenMS or LEV+EIG. However, the total processing time with RALPS can be easily reduced to less than an hour by employing four or more CPUs. We conclude that RALPS, ComBat and WaveICA showed overall competitive performance on the Cherkaoui *et al.* data. However, RALPS is the only method that combines top normalization performance with minimum biological information loss and preservation of spectral properties. A closer look at the UMAP embeddings of the normalized data produced by RALPS reveals that virtually all divisions across batches were successfully removed (Fig. 3). Similar results were obtained when applying RALPS to the full dataset by Cherkaoui *et al.*, including adducts and features with poor annotation confidence (Supplementary Fig. S7).

One important issue is the striking difference between NormAE and RALPS, which are both based on adversarial learning but yielded opposing results. In the Cherkaoui *et al.* data, NormAE reproducibly creates a collapsed solution driven by the growing classifier loss (Supplementary Fig. S8). This happens when the batches are already fairly mixed in the initial data and the classifier fails to tell them apart. The classifier loss grows and keeps contributing to the joint loss function of the autoencoder, which ultimately leads to a singular output matrix. In RALPS, this degenerate behavior is prevented by the early stopping.

### 2.6 Top performance requires three-term loss function

**Fig. 4.**
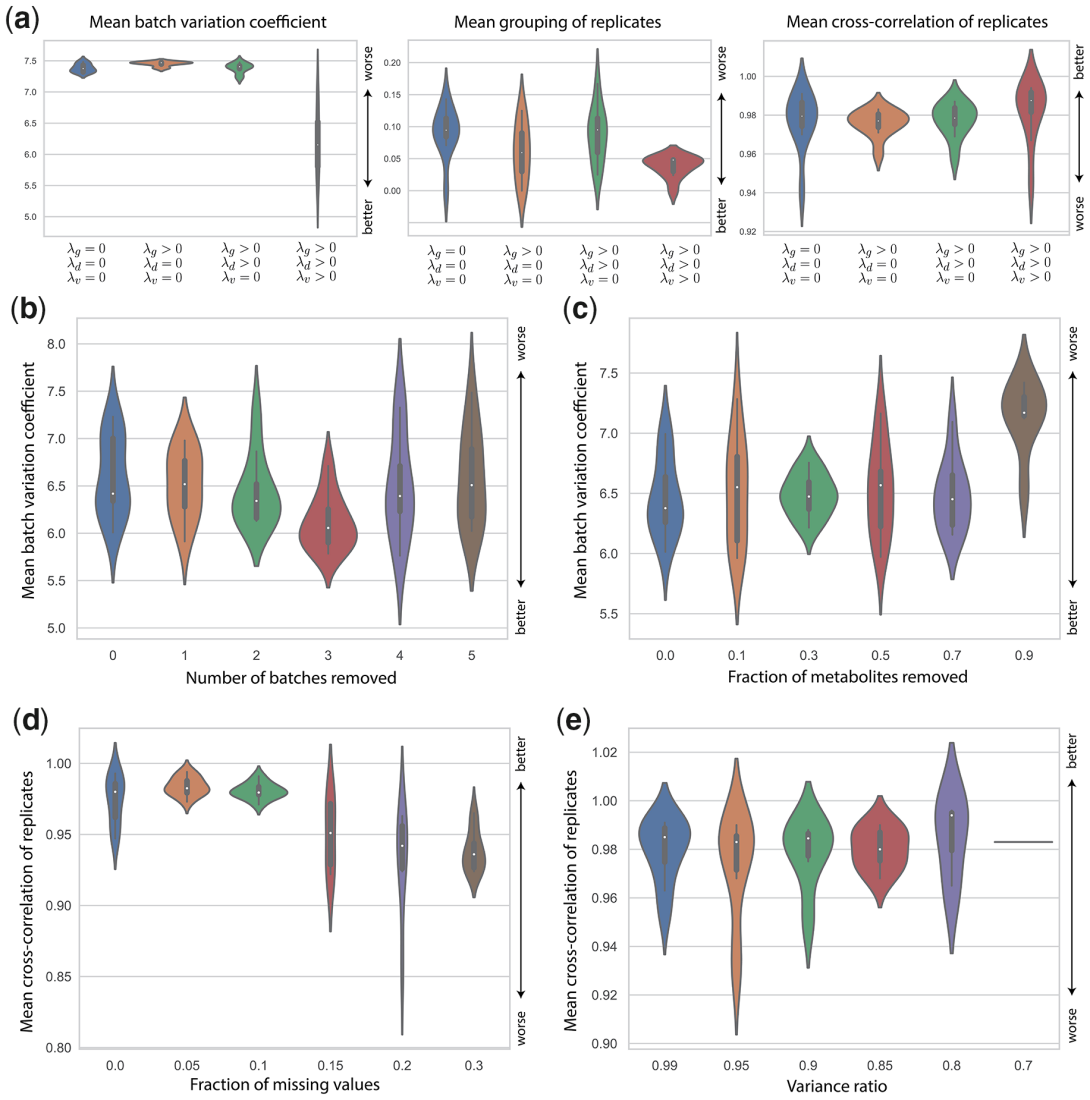
Ablation experiments. (**a**) Impact of regularization terms on key evaluation metrics. From left to right, an increasing number of terms were introduced. (**b**) Impact of the number of batches removed on the mean batch VC. (**c**) Impact of missing metabolites on the mean batch VC. (**d**) Impact of missing values on the mean cross-correlation of reference samples. (**e**) Impact of different values of *variance_ratio* parameter on the mean cross-correlation of reference samples

After demonstrating the performance of RALPS in practice, we set out to investigate in more detail how the regularization terms in the composite objective function (i.e. *L* in Fig. 1) impact the outcomes. For this investigation, we used the benchmarking dataset and two reference sample groups (as in Supplementary Fig. S3) and tested four scenarios of objective functions with 0–3 regularization terms (Fig. 4a). For example, the case of *λ*_d_ = *λ*_g_ = *λ*_v_ = 0 corresponds to an autoencoder that is solely trained to reconstruct the data without any penalty. The last scenario with non-zero *λ*_d_, *λ*_g_ and *λ*_v_ reflects the default architecture with all regularization terms. For each scenario, we applied RALPS with default parameters and performed a randomized grid search of size 100, selected the 10 top-performing normalization solutions and compared their key metrics (Fig. 4a). As expected, introducing *λ*_g_ > 0 improved tight clustering of reference samples in the embedded space. Introducing a penalty for the batch classifier with *λ*_d_ > 0 had only subtle effects on tight clustering and replicate cross-correlation. Clearly, top performance on all three criteria shown is achieved only in combination with *λ*_v_ > 0. These results indicate that the three terms in the composite objective function *L* of RALPS have a synergetic effect on batch normalization.

### 2.7 RALPS is robust against data ablations

Missing values represent a particular challenge for normalization of untargeted metabolomics data. Values are missing when they are undetectable in a subset of samples, as is common for rare compounds, such as derivatives of drugs or special food additives in human serum, or low concentration compounds near the analytical limits of detection. To verify the robustness of RALPS, we performed multiple ablation experiments. All tests were done on the benchmarking dataset using default parameters and the same two reference sample groups as above. First, we ran RALPS on subsets of the initial dataset. We shrank the dataset batch-wise from seven to two (i.e. down to 29% of the samples) keeping the chronological acquisition order. On each iteration, we removed the latest batch and applied RALPS to normalize the remaining data. We observed that the mean batch VC was generally constant and comparable to the full data case (Fig. 4b). We then shrank the dataset by removing random metabolites down to 10% of the initial number. Only in the last point, corresponding to 17 metabolites, did the mean batch variance tangibly worsen (Fig. 4c).

Next, we tested the robustness to randomly distributed missing values. A user-defined parameter (min_relevant_intensity) instructs RALPS on the lowest value to consider. The default is 1000 counts, and missing values are replaced with this minimum value. In our test, we replaced 0%, 5%, 10%, 15%, 20% and 30% randomly picked values in the data matrix with the default minimum value and then trained RALPS. We observed a decreasing trend in cross-correlation of replicates as the fraction of missing values went up (Fig. 4d). However, the decrease in mean correlation coefficients was marginal (from 0.98 to 0.94 with 30% missing values), indicating that RALPS is generally robust against missing values.

Finally, we verified how the number of neurons in model architectures affects normalization results. By default, RALPS uses as many neurons as number of principal components necessary to explain at least 90% of variance. We tested multiple values ranging from 99% to 70% variance. We did not observe any clear trends in any of four metrics used to evaluate and compare methods. The mean cross-correlation of replicates stayed above 0.98 in all cases (Fig. 4e).

## 3 Discussion

We introduce RALPS, a novel batch normalization method based on regularized adversarial learning for untargeted metabolomics data. In this work, we demonstrated its performance on two representative datasets with thousands of samples or spectral features. The benchmarking dataset was generated to test the algorithm on MS data produced over several months. In the case of the cancer cell line data by Cherkaoui *et al.*, batches instead were associated with cultivation and sampling of samples over the span of almost a year, whereas MS analysis was done sequentially with all samples. We demonstrated that RALPS outperformed other state-of-the-art methods on several key metrics. RALPS offers additional features, such as adaptive network architectures, embedded hyperparameter optimization, automated model selection and input validation. Together, these features convey flexibility, scalability, usability and robustness as confirmed by testing with different configurations of reference samples and in ablation experiments.

Historically, the loss function with three terms embedded in RALPS evolved from multiple tests, we performed with several datasets, some of which are not described here. The classification term is the component that drives the removal of batch effects. However, a deeper analysis of the resulting normalized data revealed novel problems that prompted us to introduce additional terms. The observed increase in VCs for supposedly identical samples (e.g. replicates) upon normalization is common to most methods and is a particularly acute problem for NormAE. To our best knowledge, this issue has not previously been acknowledged or addressed. We offer two hypothetical explanations for this gap. The first is the use of mean absolute error for the reconstruction loss, which is sensitive to outliers. The second is that generally increasing the noise, and thus the VC, makes it more difficult for the classifier to separate batches. Hence, there is an apparent beneficial effect on batch discrimination, but detrimental side effects in downstream analyses. Introducing the variation loss *L*_v_ mitigated but did not abolish the problem.

A closer investigation of the RALPS results revealed that the increase in VC arose from a small set of samples (1.2–1.8% of the total for the tested datasets). Filtering these samples by outlier detection (explained below) reversed the increase in VCs for RALPS and EigenMS, but not for NormAE, ComBat, WaveICA and PQN+POW (Supplementary Fig. S9), suggesting that the latter methods produced even more outliers. We do not advocate for outlier detection and removal or a particular approach for it, but we recommend that researchers using batch normalization methods carefully evaluate sample-wise VCs and consider correcting them before performing downstream statistical analysis.

The grouping regularization term *r*_g_ was the second novel addition to the adversarial training and the key to preserving similarity of supposedly equal samples. Although RALPS relies on clustering of reference samples in the embedded space to assess grouping, alternative approaches could be considered. For example, *r*_g_ could be calculated from distances in the latent space. Distance-based metrics would carry several hypothetical advantages and pitfalls. Owing to the fast computation, training would be tangibly shorter than with clustering. We expect that distance-based metrics would perform better with data characterized by subtle batch effects in which clustering fails to separate reference samples. Even in the case of all replicates falling into a single cluster (which happens naturally when most batch effects have been already removed), minimizing distances between all pairs of replicates would still have a regularization effect, whereas clustering would not. Among the potential drawbacks, we would expect an increased sensitivity to single outliers. Alternative paradigms for similarity preservation should be tested in the future. One additional aspect that could be optimized is the clustering algorithm. RALPS includes six methods, and HDBSCAN was chosen as the default based on performance, robustness and speed (Supplementary Fig. S10). The outcome might differ for different experiments.

In terms of experimental design, RALPS requires reference samples across batches, but it is not strictly necessary to have the very same reference samples present in all batches. We illustrated this flexibility in our normalization of the Cherkaoui *et al.* data, in which two reference sample groups were present in only four and five of the seven batches, respectively. This feature leaves considerable freedom for the experimental design, in particular for the number of replicate groups to include. Based on our tests with the benchmarking dataset and other studies not shown here, using replicates from a pooled study sample in each batch is generally sufficient to correct for typical batch biases arising from untargeted metabolomics measurements performed over different days. If the total number of samples does not become prohibitively large, including a second group of reference samples is beneficial. In this scenario, the recommendation is to spike in a reduced set of compounds of interest and at low concentrations to present a realistic challenge for calculating the grouping term *r*_g_. In contrast, blanks do not constitute a good control group because they can readily be distinguished from all other samples and are ineffective in the training process. The flexibility of RALPS allows it to be tested on existing datasets or applied in experiments that were not specifically designed with its use in mind. In all cases, RALPS is developed to make full use of all available reference samples in correcting inter-batch experiments.

In the case of multi-batch experiments that are affected both by inter and intra-batch issues, such as excessive sample-to-sample variability or temporal drifts, we recommend a two-step procedure. First, all single batches should be corrected individually to maximize coherence within each individual batch. Second, RALPS should be applied to harmonize data across batches.

Finally, we see no fundamental problem in using RALPS to normalize any kind of tabular data consisting of samples coming from different batches and with features characterizing the samples. Information about the similarity of samples (for references and beyond) can be encoded via groups in the batch information file. We encourage researchers from other omics fields to challenge RALPS with their own data and drive its further development by reporting experience and issues in the repository.

## Supplementary Material

btad096_Supplementary_Data
