# Supplementary material for: Regularized adversarial learning for normalization of multi-batch untargeted metabolomics data
Source: Bioinformatics. 2023 Feb 24;39(3):btad096. doi: 10.1093/bioinformatics/btad096 (PMC9978579; doi:10.1093/bioinformatics/btad096)
Supplement: btad096_Supplementary_Data [file btad096_supplementary_data.pdf]

# Supplementary Methods, Figures, and Tables for

## Regularized adversarial learning for normalization of multi-batch untargeted metabolomics data

Andrei Dmitrenko[1,2,3], Michelle Reid[1], Nicola Zamboni[1,3]*

[1] Institute of Molecular Systems Biology, Department of Biology, ETH Zürich, CH-8093 Zürich, Switzerland

[2] Life Science Zurich PhD Program on Systems Biology

[3] PHRT Swiss Multi-OMICS Center, Switzerland

# Supplementary Methods

## Generation of a multi-batch benchmarking dataset

We assembled a panel of 136 samples with a large variety in sample type, complexity, and concentrations (**Supplementary Figure 1**). To ensure a fair representation of complex samples, we prepared roughly half of them using human serum extracts (NIST SRM1950). We included spike-ins with selections of amino acids, fatty acids, and nucleobases, and a fully $^{13}$C-labeled *E. coli* extract at different dilutions. The samples were distributed on two 96-well plates. Both plates were replicated several times and stored at –80 °C. The two plates were repeatedly analyzed in independent batches by untargeted metabolomics using flow injection–time-of-flight (TOF) MS on the same Agilent 6550 iFunnel Q-TOF system and in negative mode ionization[14]. The measurement of the 136 samples in technical triplicates and hundreds of intercalated blanks took ca. 12 hours. The same sequence was analyzed seven times over the span of about 2 months with time gaps of up to 3 weeks. Between batches, the instrument was used in different experiments and underwent routine maintenance as well as tuning procedures. From an analytical standpoint, all batches were acquired with an instrument that was operating normally, i.e., without any indication of problems that might affect measurement quality.

Each batch was analyzed independently. To focus on the subset of m/z peaks that possibly relate to metabolites, we selected features with m/z values that were matched to deprotonated compounds listed by the Human Metabolome Database (ver. 4.0, tolerance 0.001 Da). We intersected the putative peak lists obtained from each batch by m/z and retained those features that could be reproducibly detected. Low abundance ions with average intensity < 1000 counts were filtered. This procedure resulted in a consolidate data table with intensities for 170 putative deprotonated metabolites and 2856 files, divided in seven batches.

## Test data

The full multi-batch benchmarking dataset is available at https://doi.org/10.3929/ethz-b-000545373. The filtered data table used in our experiments is provided with the code. The full cancer metabolomics dataset by Cherkaoui et al. is available at https://doi.org/10.3929/ethz-b-000511784.

## Loss functions and optimizers

RALPS uses mean square error loss for the autoencoder and cross-entropy loss for the classifier. Mean square error was preferred over mean absolute error because of faster convergence and better reconstruction quality when trained without the classifier. Consequently, RALPS does not use pretraining epochs; both model networks are trained in turns from the start. Adam optimizer is used for both.

## Regularization term preserving similarity of samples

We introduce a regularization term $r_g$ to preserve the similarity of reference samples while running an adversarial training loop. This term explicitly takes into account the grouping of reference samples across batches in the embedded space (**Figure 1**). At every training

epoch, the representations of the input data are clustered, and the grouping coefficient for each reference sample is computed based on how many replicates of the sample across batches appear to be in the same cluster. The regularization term penalizes the reconstruction loss ($r_g > 0$) when the replicates of the reference samples happen to be in different clusters. Conversely, the regularization cancels out ($r_g = 0$) when reference samples across batches are clustered together. By design, the regularization term $r_g$ is limited from above ($r_g \leq 1$). Before clustering, RALPS uses the UMAP algorithm to generate embeddings of the learned representations. Six clustering algorithms are implemented in RALPS: HDBSCAN (default), UPGMA, Mean-shift, OPTICS, BIRCH, and Spectral clustering. By default, the minimum cluster size is the number of batches multiplied by the number of replicates in the data. Single clusters are not allowed.

## Adaptive network architecture

Although the backbones of the architectures for the autoencoder and the classifier are fixed, the number of neurons in some of their layers is automatically adjusted to the data. Principal component analysis is used to find the number of components that capture a user-defined percent of variance (selected randomly among 90%, 95%, or 99% by default). The number determines the dimensionality of the autoencoder bottleneck layer and the classifier input layer. Thereby, RALPS adapts to datasets of different sizes and variances.

## Randomized grid search of hyperparameters

A few hyperparameters such as learning rates, regularization coefficients, batch size, and minimal variance ratio for principal component analysis are required to run RALPS. If the user does not provide them, they are sampled randomly from the predefined ranges. This is typically the case for the six parameters related to learning rates and loss coefficients. We also implemented ways for users to define their own ranges of hyperparameters for sampling. Every unique parameter set is assigned its own unique ID that is used to name a directory in the file system, where all the corresponding results will be stored. The user also provides the size of the randomized grid (defaults to 1, a single run). Beyond exploring the hyperparameter space and finding the best normalization solutions, such implementation allows for convenient testing of model robustness. Fixing training hyperparameters and setting the randomized grid size to values >1 facilitates investigations of how randomization during training affects performance of a particular parameter set. More information on the hyperparameters and the configuration file is provided on the github repository.

## Model selection

Several metrics are tracked during the training process to evaluate model performance and select the best model: (i) grouping coefficient calculated on the clustering results, (ii) statistic reflecting cross-correlation of replicates across batches, (iii) mean batch VC, and (iv) percent of samples with increased VCs. These metrics are calculated at each epoch for the two sample types: the reference samples used for $r_g$ regularization, and for benchmarking

samples if specified by the user. Defining benchmarking samples is optional but may be useful to verify that the normalization solution is not overfitted to the reference samples.

Within a single run, the best model (epoch) is supposed to achieve low grouping coefficient of reference samples, high cross-correlation, reduced mean VC of the reference samples, and reduced mean batch VC. The best epoch is selected after the training is complete based on the reference samples and the overall reconstruction loss. First, the epochs with 10% of the lowest grouping coefficients are selected. Among these epochs, the 10% of the highest cross-correlation is selected. Among the remaining epochs, the best one corresponds to the minimal reconstruction loss. To avoid the risk of exploding classifier loss and the over-normalization problem, we implemented an early stopping criterion that interrupts training if the classifier loss starts continuous growing. Such models are marked as stopped early. An exemplary run is illustrated in **Supplementary Figure 10**.

Model selection for the randomized grid search applies to the best epochs selected previously for each parameter set. The best models across all parameter sets are selected using a similar logic:

1. The lowest mean batch VC and the highest cross-correlation values.
2. The lowest grouping coefficient and the highest cross-correlation values.
3. The lowest grouping coefficient and the lowest percent of increased VCs.

The models corresponding to these groups are combined and sorted by the reconstruction loss. The first 10 models are marked as the best in the randomized grid search output file containing all metrics of all evaluated parameter sets. Based on tests with multiple data sets, we recommend using a *grid_size* ≥ 50 to have high chances of finding a (nearly) optimal normalization solution. This heuristic model selection strategy proved to be sufficient to pick the models of the best normalization effects in all applications presented in this study. However, RALPS keeps all model results so that the user can select a different solution, based on a combination of quantitative and qualitative criteria available for each model.

## Outlier detection

In the attempt to control for increased VCs, we adopted a version of a boxplot outlier removal approach. For each sample in the normalized data, we removed metabolites with intensities below $Q1 - \alpha \cdot IQR$ or above $Q3 + \alpha \cdot IQR$, where $Q1$ is the 25$^{th}$ intensity percentile, $Q3$ is the 75$^{th}$ percentile, $IQR$ is the interquartile range, and $\alpha$ is a parameter (defaults to 1.5 for a classical boxplot). The script is available in the code repository.

For each application of RALPS described earlier in this paper, we selected $\alpha$ such that only 1% of samples had increased VCs. After filtering the normalized data, we calculated how many metabolites were dropped and then applied exactly the same procedure to the data normalized by other methods to compare their propensity for generating outliers.

# Supplementary Figures

**Supplementary Figure 1. Description of the benchmarking dataset.** The dataset is composed of 136 samples distributed on two 96-well plates. On each plate, rows are organized as a dilution series of one of the following samples: (S) a methanol extract of the NIST SRM1950; (AA) an amino acid mix with glycine, proline, asparagine, lysine, phenylalanine, and tyrosine; (FA) a fatty acid mix with lauric acid, palmitic acid, and stearic acid; (PP) a mix of nucleobases with cytosine, uracil, thymine, adenine, and guanine; (AA+FA+PP): a mix of the three mixes; (13C): a polar metabolic extract of fully 13C-labeled *Escherichia coli*. Rows B-F on Plate 2 include a constant amount of SRM1950 extract. The amounts of compounds in the standard mixes were empirically optimized such that about four dilutions are detectable on top of undiluted SRM. From columns A to H, samples were diluted in eleven two-fold steps. Therefore, column H is a 2048-fold dilution of column A. The samples labels indicate the plate number followed by the content and the dilution factor.

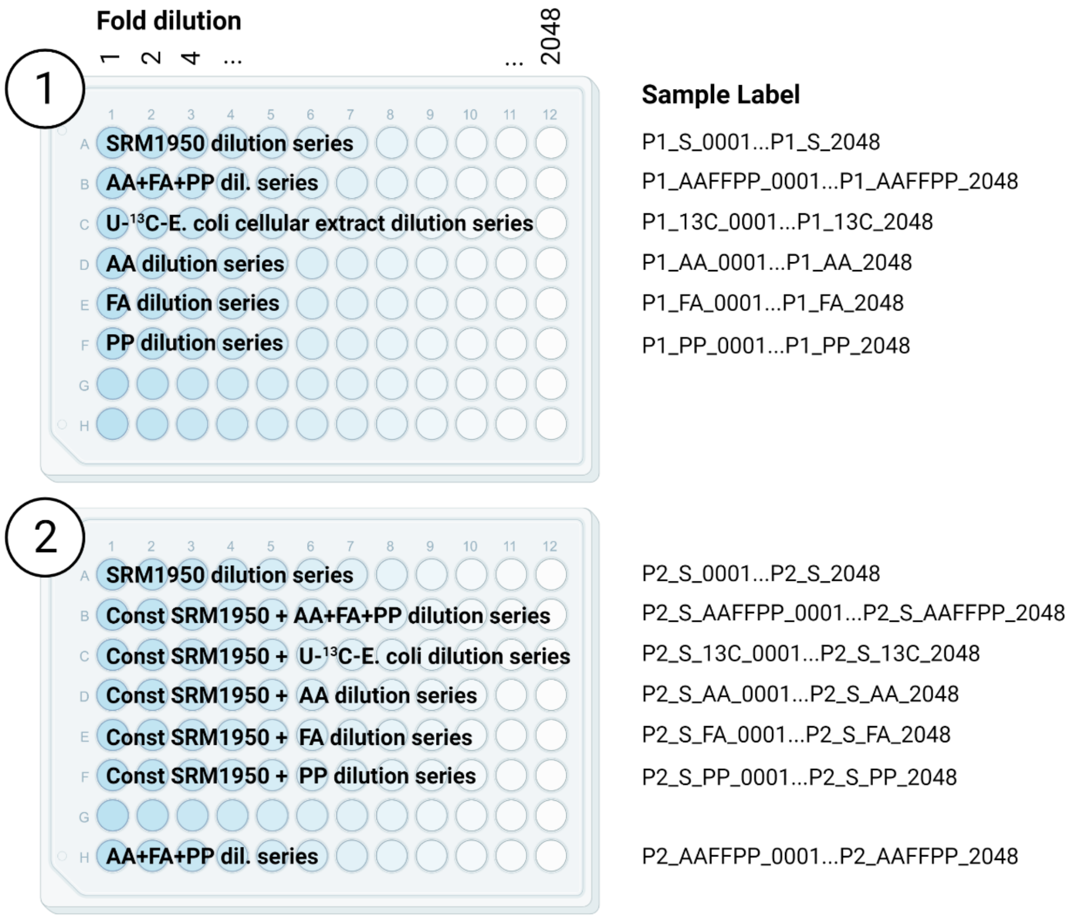

**Supplementary Figure 2. Application to the benchmarking dataset using all study samples as references.** (**a, d**) UMAP embeddings. (**b, e**) Distributions of cross-correlations. (**c, f**) Mean batch variation coefficients. Left panels refer to the initial data, right panels to the normalized data produced by RALPS.

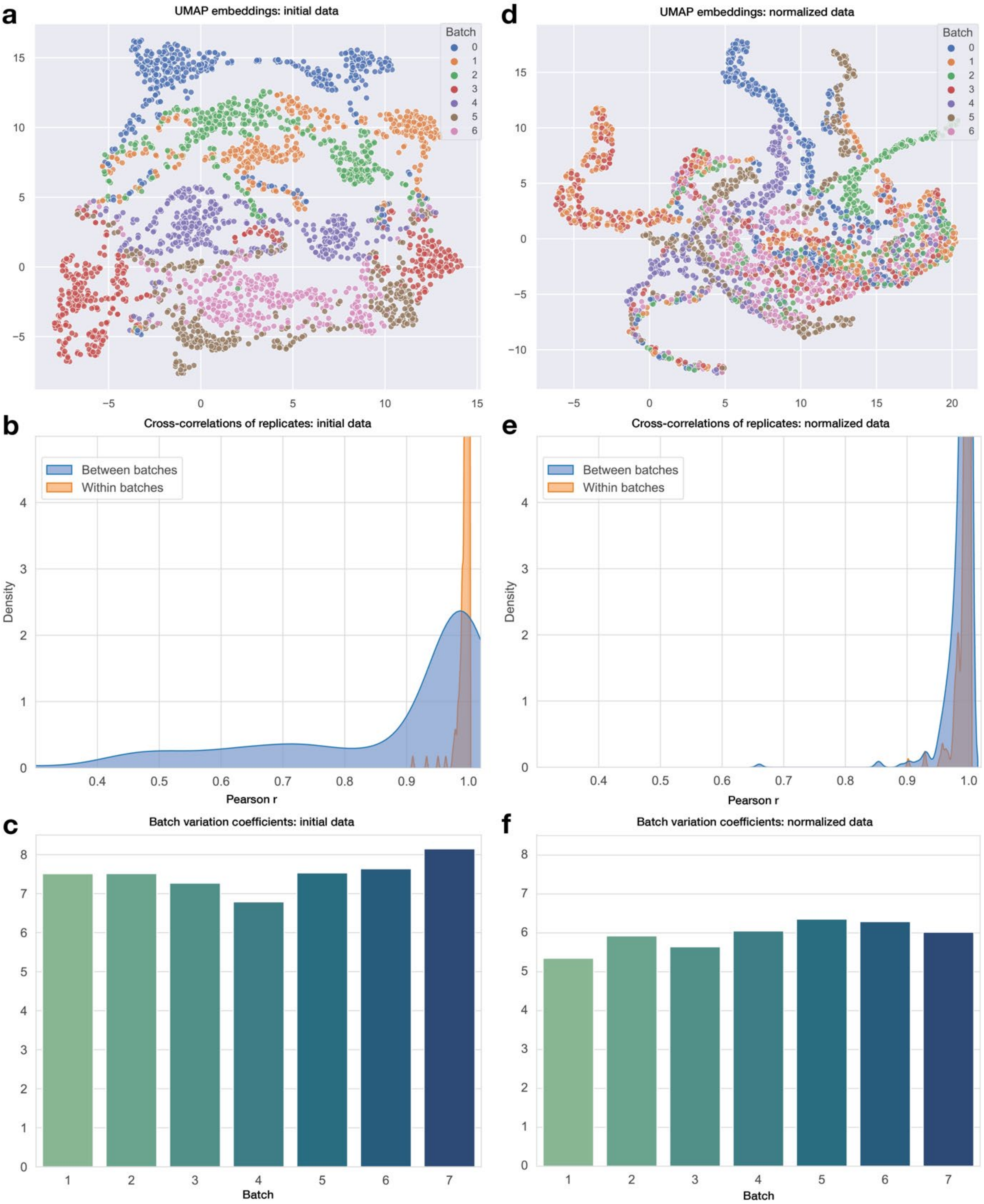

**Supplementary Figure 3.** UMAP embeddings of the initial (left) and normalized (right) data using P2_S_0001 and P2_S_PP_0001 as reference samples.

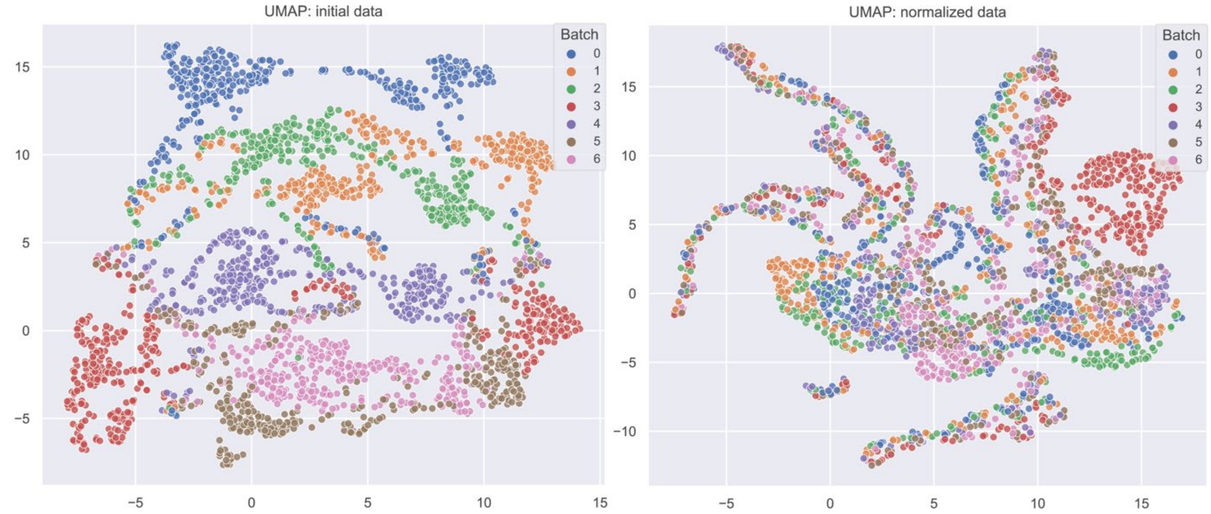

**Supplementary Figure 4**. (**a**) UMAP embeddings of the initial data. (**b-f**) UMAP embeddings of the data normalized with reference samples groups listed in **Supplementary Table 1**. Note that the snake-like patterns are associated to dilution series present in the benchmarking data set.

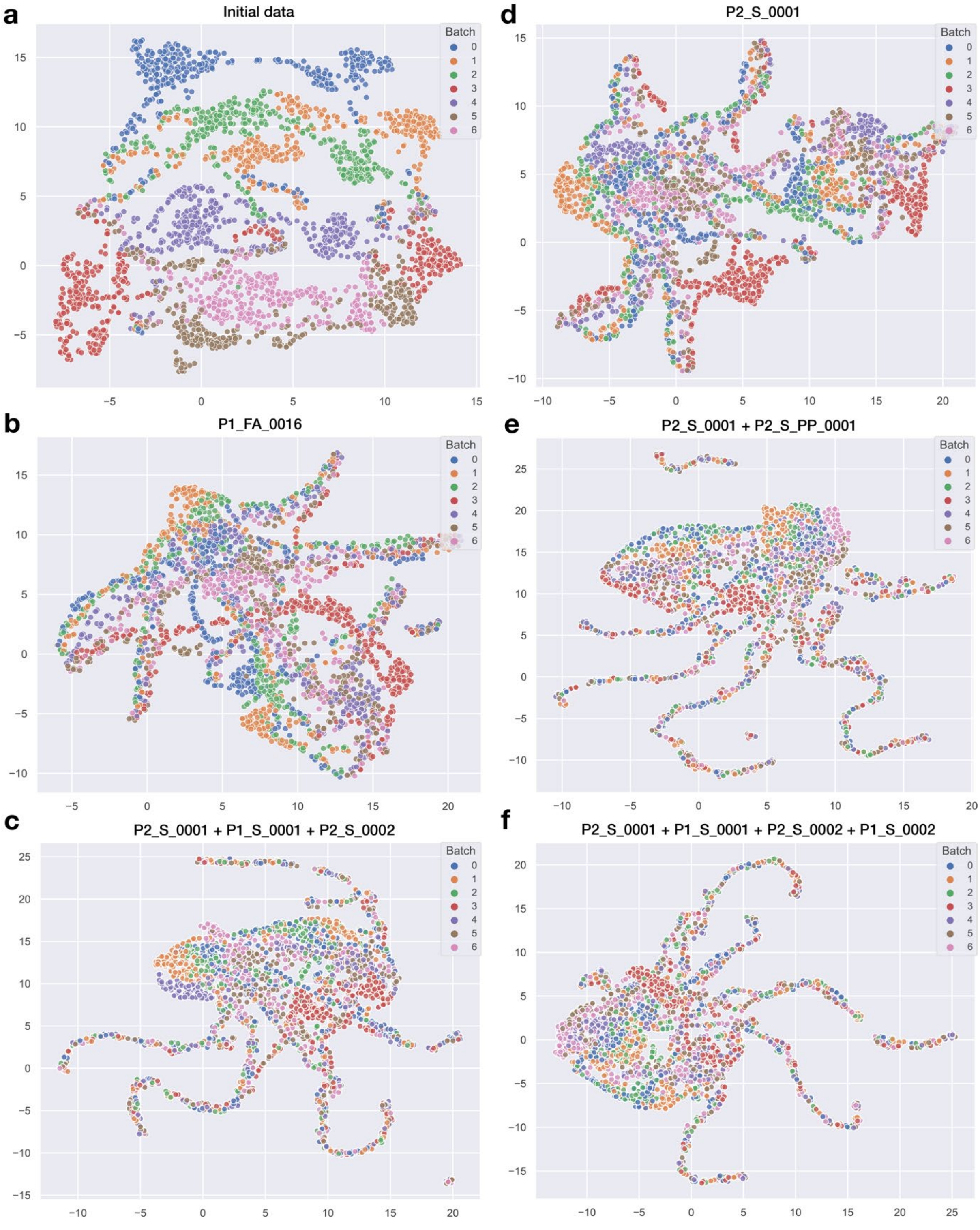

**Supplementary Figure 5**. Cross-correlation heatmaps for replicate samples of fatty acids sample with 8x dilution (P1_FA_0008).

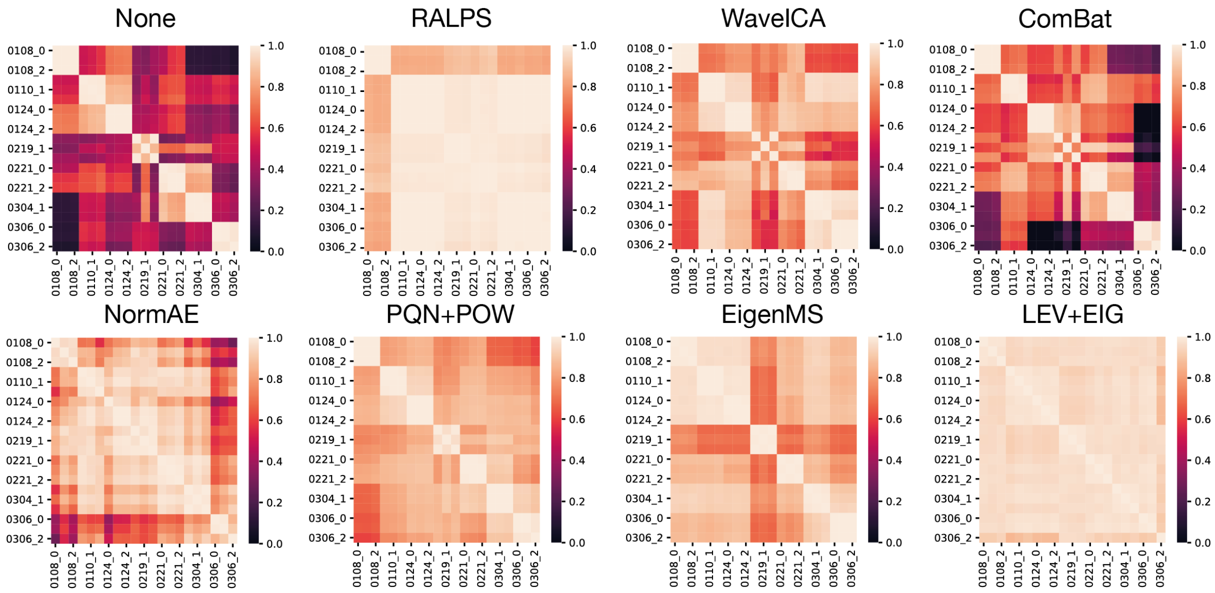

**Supplementary Figure 6**. Comparison of methods for the cancer cell lines dataset by Cherkaoui et al. (**a**) Cross-correlation heatmaps for MDAMB231 replicates. (**b**) Full spectra. (**c**) Percent of samples and feature combination with increased variation coefficients. (**d**) Mean batch variation coefficient.

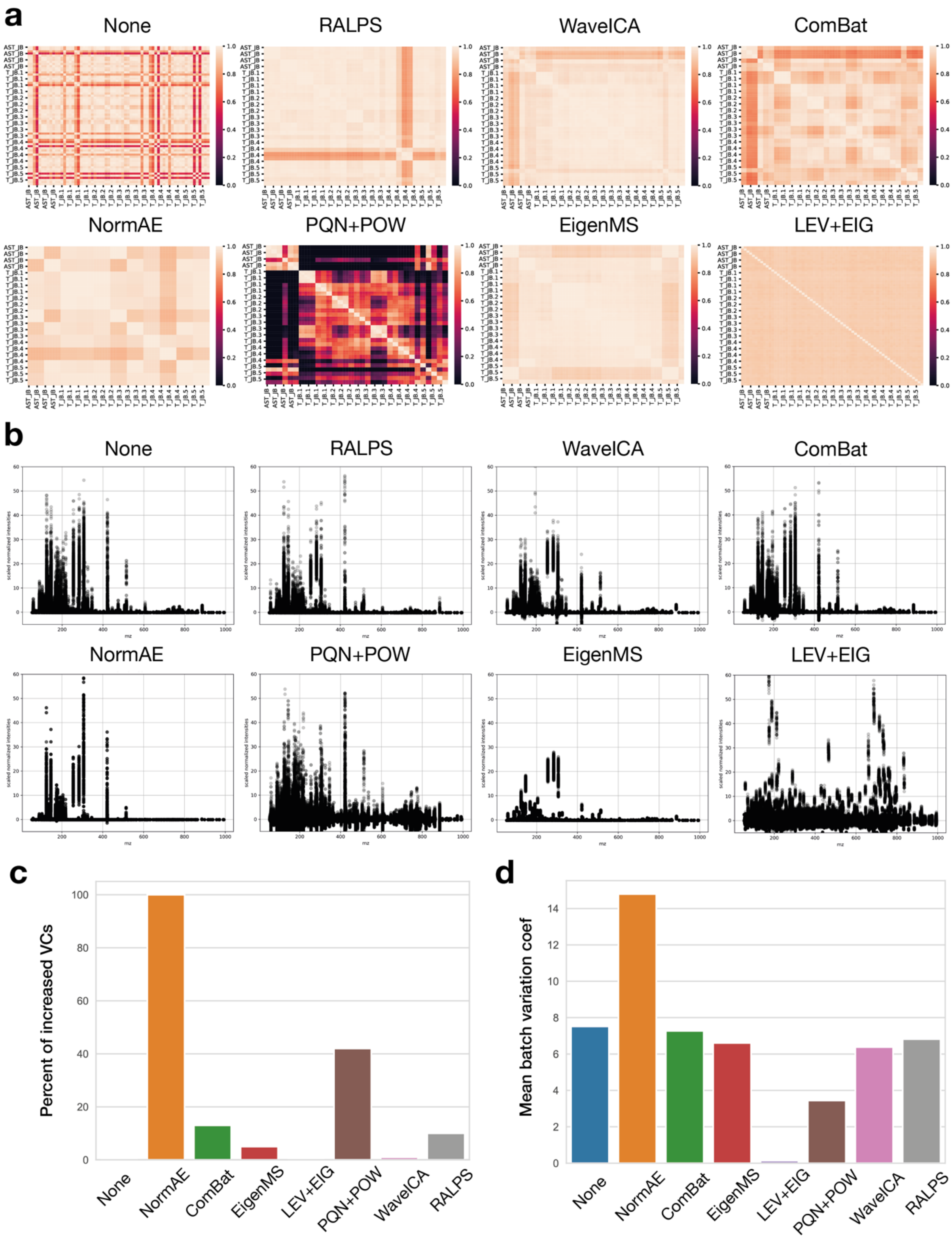

**Supplementary Figure 7.** Performance of RALPS on the full Cherkaoui et al. data including adducts (1289 features x 2904 samples in 8 batches). Cross-correlations are reported for a representative QC sample (P1 S 0001) which was not used for training. Upon normalization, within and between correlations are > 0.95, with a unique outlier that had a correlation close to zero in the initial data and ~0.7 upon normalization. RALPS performance is consistent also in the presence of adducts and less confident annotations.

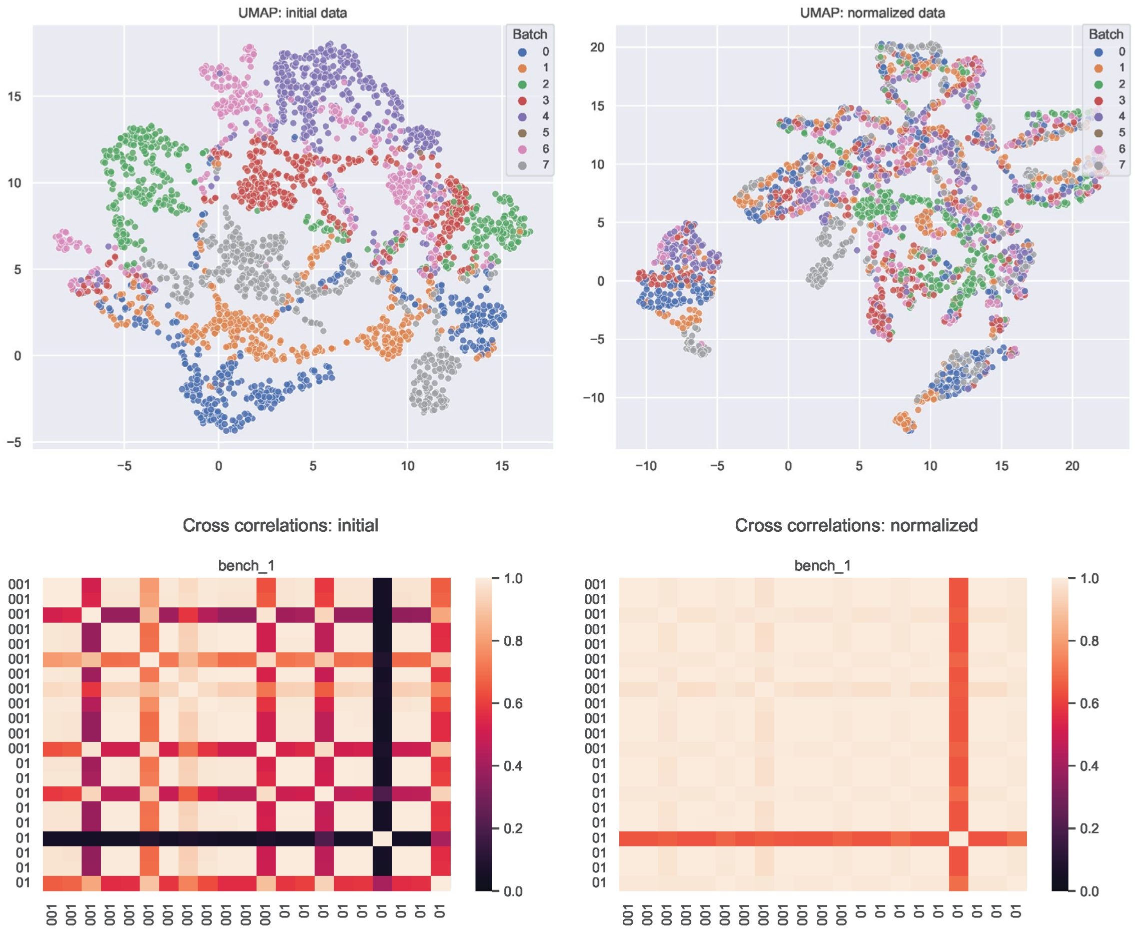

**Supplementary Figure 8**. Training losses plot generated by NormAE. (**a**) The first 1000 epochs are used for the autoencoder pretraining without the classifier. Then, the adversarial training epochs follow, where the autoencoder and batch classifier are trained in turns. We observe a fast decrease in the classifier loss followed by an "explosion". Further training leads to the collapsed solution. Percent of unique values in MDAMB231 reference sample before and after normalization. (**b**) We observe that NormAE normalized the data such that it contains less than 20% of unique values. This indicates over-normalization, i.e., most of the biological samples became numerically identical and, therefore, non-comparable among each other.

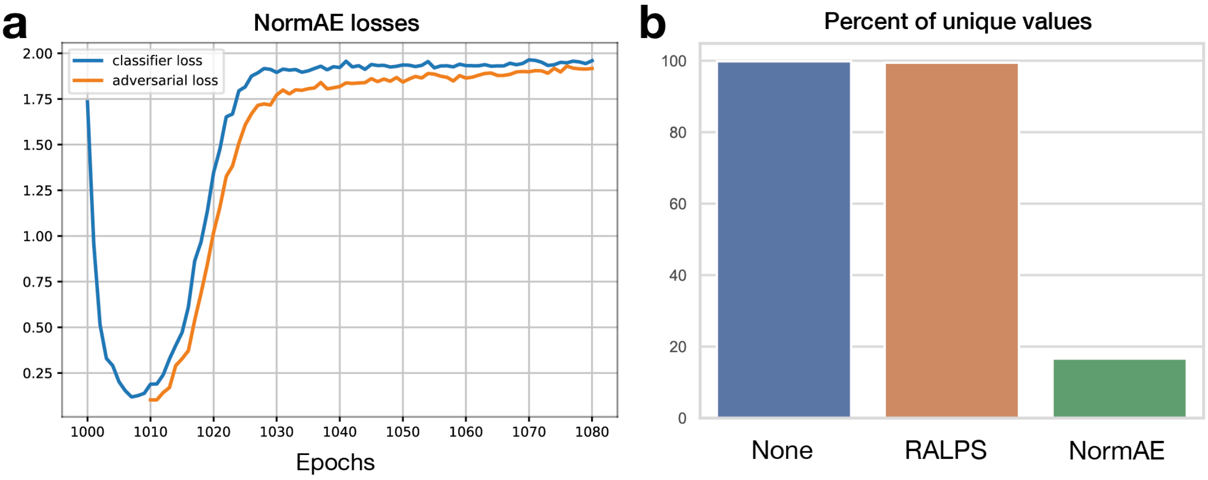

**Supplementary Figure 9. Outlier detection and removal.** (**a**) Percent of samples with increased VCs after filtering. (**b**) Number of metabolites removed during filtering. The results were obtained with the same outlier removal procedure applied to the benchmarking dataset after normalization.

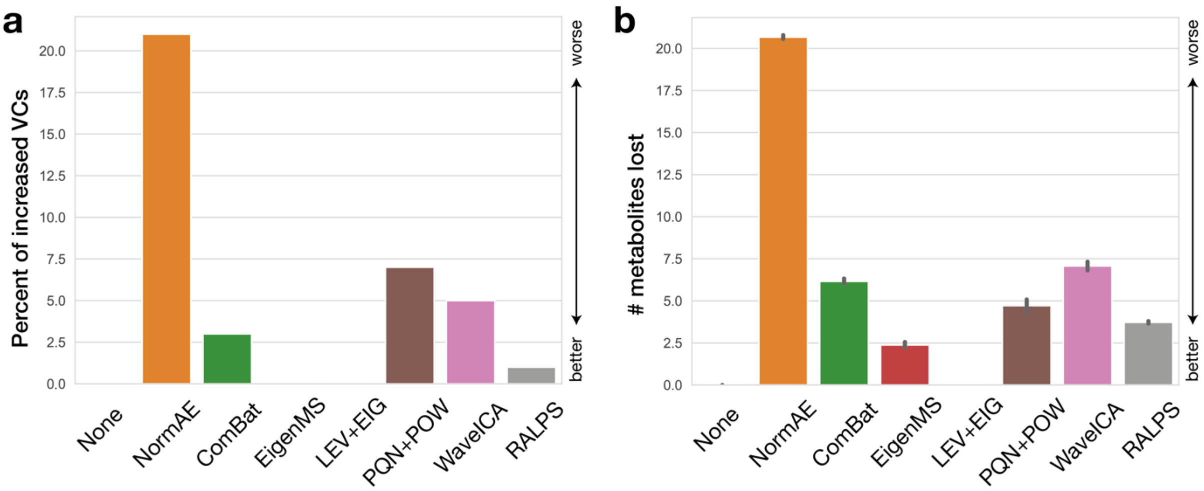

**Supplementary Figure 10**. **Comparison of clustering algorithms for the normalization of the benchmarking dataset.** Six clustering algorithms are implemented in RALPS and are compared across metrics. (**a**) Mean cross-correlation of replicates. (**b**) Mean batch variation coefficient. (**c**) Mean grouping coefficient for samples' replicates. (**d**) Mean total RALPS runtime.

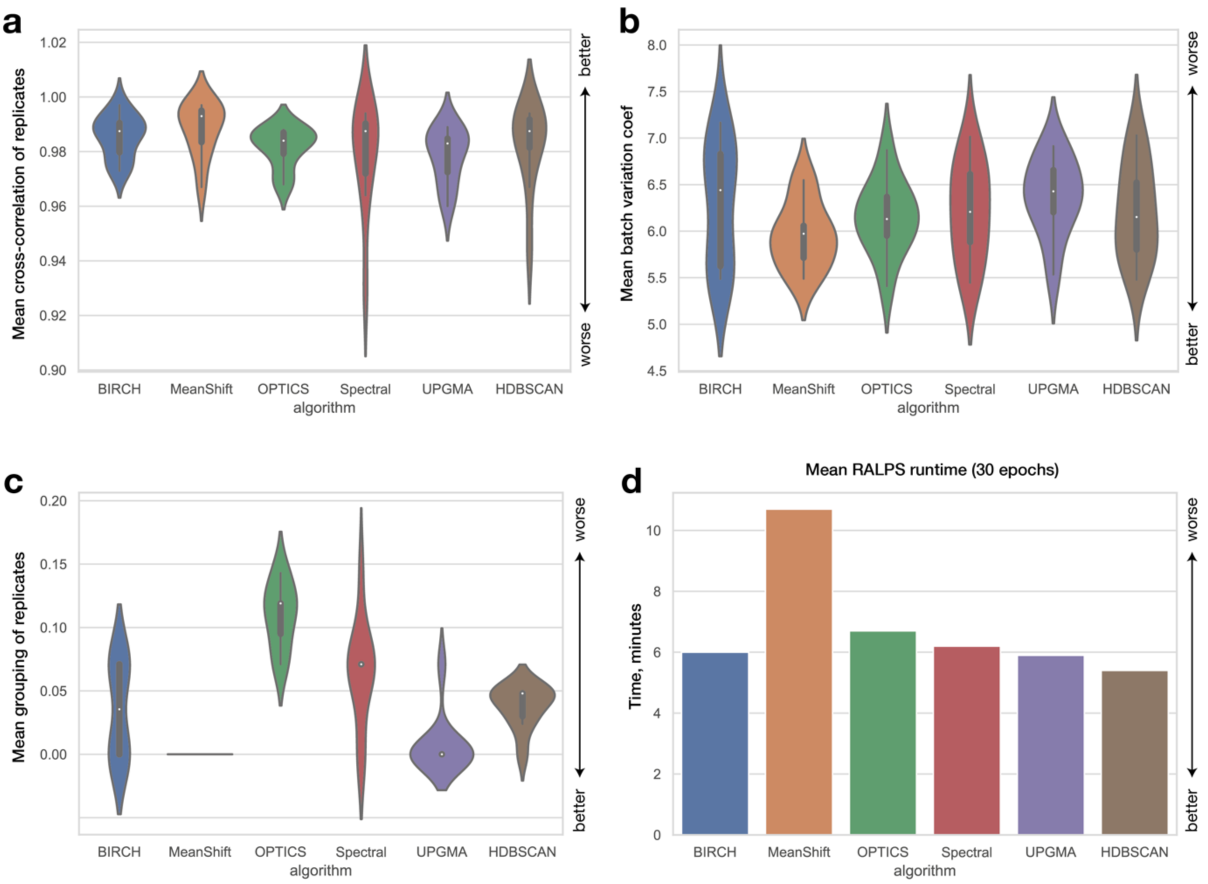

# Supplementary Tables

**Supplementary Table 1. Best normalization solutions of different reference samples.** Autoencoder reconstruction loss, grouping (*the lower the better*) and Pearson correlation (*the higher the better*) coefficients are presented for samples' replicates. A solution was treated reproducible if at least three other solutions with comparable could be found in an experiment of repetitive training with the same parameter set.

| Test # | Reference samples | reproducible | MSE loss | grouping | correlation |
|---|---|---|---|---|---|
| 1 | P2_S_0001<br>P2_S_PP_0001 | Yes | 3.097 | 0.0 | 0.969 |
| 2 | P2_S_0001 | No | 2.385 | 0.0 | 0.969 |
| 3 | P2_S_0001<br>P2_S_FA_0001 | No | 1.632 | 0.071 | 0.967 |
| 4 | P2_S_0001<br>P2_SF_0001 | No | 2.028 | 0.119 | 0.942 |
| 5 | P2_S_0001<br>P2_S_0002<br>P2_S_0004 | Yes | 2.241 | 0.0 | 0.961 |
| 6 | P2_S_0001<br>P1_S_0001<br>P2_S_0002 | Yes | 3.684 | 0.033 | 0.972 |
| 7 | P2_S_0001<br>P2_S_0002<br>P2_S_0004<br>P2_S_0008 | Yes | 2.190 | 0.094 | 0.972 |
| 8 | P2_S_0001<br>P2_S_0002<br>P2_S_PP_0001<br>P2_S_PP_0002 | No | 1.775 | 0.113 | 0.955 |
| 9 | P2_S_0001<br>P1_S_0001<br>P2_S_0002<br>P1_S_0002 | Yes | 2.358 | 0.0 | 0.970 |
| 10 | P1_FA_0016 | No | 17.278 | 0.095 | 0.910 |

**Supplementary Table 2**. **Ion intensity ranges of cancer cell line data by Cherkaoui et al.** Only methods that output ion intensities are given.

| method | min | max | negative values |
|---|---|---|---|
| None | 0 | 125,663,017 | 0.0% |
| ComBat | - 4,354,927 | 71,056,531 | 1.0% |
| EigenMS | 402 | 28,265,811 | 0.0% |
| WaveICA | - 15,200,577 | 121,753,507 | 1.2% |
| RALPS | 0 | 95,519,700 | 0.0% |

**Supplementary Table 3**. **Run time for normalization of cancer cell line data by Cherkaoui et al.** All methods, except NormAE, were run on 2.2 GHz Intel Core i7 8750H (“Coffee Lake”) under Mac OS Big Sur. * NormAE was run on a Nvidia GeForce RTX 2060 under Windows 10.

| | LEV+EIG | PQN+POW | ComBat | EigenMS | WaveICA | NormAE | RALPS |
|---|---|---|---|---|---|---|---|
| Time (min) | 127 | 6 | 1 | 111 | 5 | 136* | 3 |
